# The 2022 dengue outbreak in Bangladesh: hypotheses for the late resurgence of cases and fatalities

**DOI:** 10.1093/jme/tjad057

**Published:** 2023-05-18

**Authors:** Najmul Haider, Mohammad Nayeem Hasan, Ibrahim Khalil, Daniel Tonge, Shivanand Hegde, Muhammad Abdul Baker Chowdhury, Mahbubur Rahman, Manjur Hossain Khan, Rashid Ansumana, Alimuddin Zumla, Md Jamal Uddin

**Affiliations:** School of Life Sciences, Faculty of Natural Sciences, Keele University, Staffordshire ST5 5BG, UK; Department of Statistics, Shahjalal University of Science and Technology, Sylhet 3114, Bangladesh; Department of Livestock Services, Ministry of Fisheries and Livestock, Dhaka, Bangladesh; School of Life Sciences, Faculty of Natural Sciences, Keele University, Staffordshire ST5 5BG, UK; School of Life Sciences, Faculty of Natural Sciences, Keele University, Staffordshire ST5 5BG, UK; Department of Neurosurgery, University of Florida College of Medicine, Gainesville, FL 32610, USA; Department of Pathobiology and Population Sciences, The Royal Veterinary College, University of London, Hawkshead Lane, North Mymms, Hatfield, Hertfordshire, UK; Institute of Epidemiology, Disease Control and Research (IEDCR), Ministry of Health and Family Welfare, Mohakhali, Dhaka, Bangladesh; Institute of Epidemiology, Disease Control and Research (IEDCR), Ministry of Health and Family Welfare, Mohakhali, Dhaka, Bangladesh; Department of Community Health and Clinical Studies, School of Community Health Sciences, Njala University, Bo City, Sierra Leone; Division of Infection and Immunity, Centre for Clinical Microbiology, University College London and NIHR-BRC, University College London Hospitals, London, UK; Department of Statistics, Shahjalal University of Science and Technology, Sylhet 3114, Bangladesh; Department of General Educational and Development, Daffodil International University, Dhaka, Bangladesh

**Keywords:** 2022 dengue outbreak, Bangladesh, rainfall, serotype, DENV-4

## Abstract

Bangladesh reported the highest number of annual deaths (*n* = 281) related to dengue virus infection in 2022 since the virus reappeared in the country in 2000. Earlier studies showed that >92% of the annual cases occurred between the months of August and September. The 2022 outbreak is characterized by late onset of dengue cases with unusually higher deaths in colder months, that is, October–December. Here we present possible hypotheses and explanations for this late resurgence of dengue cases. First, in 2022, the rainfall started late in the season. Compared to the monthly average rainfall for September and October between 2003 and 2021, there was 137 mm of additional monthly rainfall recorded in September and October 2022. Furthermore, the year 2022 was relatively warmer with a 0.71°C increased temperature than the mean annual temperature of the past 20 yr. Second, a new dengue virus serotype, DENV-4, had recently reintroduced/reappeared in 2022 and become the dominant serotype in the country for a large naïve population. Third, the post-pandemic return of normalcy after 2 yr of nonpharmaceutical social measures facilitates extra mosquito breeding habitats, especially in construction sites. Community engagement and regular monitoring and destruction of Aedes mosquitoes’ habitats should be prioritized to control dengue virus outbreaks in Bangladesh.

## Introduction

Dengue virus is endemic in Bangladesh and cases have been reported every year since 2000. Most of the dengue cases in Bangladesh are recorded in major cities, with more than 92% of annual cases reported between the months of August and September (Sharmin et al. 2018). This higher number of cases in these 2 months is a result of mosquito breeding which favors heavy rainfall and warmer temperatures (World Bank 2021). Studies using dengue patients’ data between 2008 and 2019 showed that the incidence of dengue cases in Bangladesh usually starts to increase in April and reaches a peak in July, before sharply declining after August (Ahsan et al. 2021, Haider et al. 2021). Although the cases reach their peak in July, the monthly growth factor (the ratio at which dengue cases change in a month compared to the previous month) reaches its peak earlier in June(Haider et al. 2021). The monthly growth factor remains above one for only 4 months: April to July (Haider et al. 2021). As of 31 December 2022, the country reported 281 dengue-related deaths which is the highest death toll due to the dengue virus in the country since 2000. In 2022 the monthly highest number of dengue cases was recorded in October (21,932 cases) and the highest number of deaths was recorded in November (113 deaths), both figures are very unusual considering the epidemiological trend of the virus in the country (Haider et al. 2021).

Dengue virus is transmitted between humans through Aedes mosquitoes: *Aedes aegypti* and *Aedes albopictus*. Both species of mosquitoes are distributed across Bangladesh (Rahman et al. 2021). There are 4 distinct serotypes of dengue virus (DENV): DEN1-DEN4 with a potential new serotype DENV-5 reported from Malaysia (Normile 2013, Muraduzzaman et al. 2018, Hamel et al. 2019). Infection by one serotype does not protect an individual from infection by another DENV serotype(Muraduzzaman et al. 2018, Shirin et al. 2019). The second infection caused by a different serotype could lead to secondary dengue infection which is often severe and can lead to dengue hemorrhagic fever (Schilling et al. 2004).

In 2019, Bangladesh reported the highest number of dengue cases (101,354 cases), and the possible drivers of the outbreak were described as the introduction of a new serotype DENV-3, unusually prolonged wet season, and resistance to insecticides used by the local authorities (Ahsan et al. 2021). During the years 2020 and 2021 with COVID-19 pandemic-related lockdown measures in place, the country experienced a relatively lower number of dengue cases (1,405 and 28,429 cases respectively). The objective of this study is to present a hypothesis and explain possible drivers of the late resurgence of dengue cases and deaths in 2022 in Bangladesh.

## Methods

We collected meteorological data including 3-hourly temperature and daily rainfall data from 2003 to 2022 from the Bangladesh Meteorological Department. The data were recorded in Mirpur Weather Station in Dhaka (N 23°46ʹ, E 90°23ʹ). We also collected monthly reported dengue cases from the Ministry of Health and Family Welfare’s (MOHFW) Directorate General of Health Service (DGHS) website (Directorate General of Health Services (DGHS) 2022). We plotted monthly rainfall, dengue cases, and death records for the period 2003–2021 and 2022 separately to understand the role of weather in the late re-surgent of dengue cases in 2022.

## Results and discussions

As of 2022 31 December, a total of 62,382 dengue cases have been recorded in Bangladesh including 21,932 cases in October alone, which is the highest number of recorded cases for this month since 2000. The country recorded 113 deaths caused by dengue virus infection in November, which is the highest number of recorded monthly deaths since 2000. In December 2022, Bangladesh recorded a total of 27 dengue-related deaths, compared to the monthly average of 0.47 deaths in December for the period 2003–2021. We present 3 possible explanations for the 2022 late-resurgent of dengue virus cases and deaths in Bangladesh: (a) the late start of the rainy season, (b) the introduction of a new serotype (DENV-4) in the country, (c) returning to normalcy after 2 yr of pandemic-related control measures has facilitated mosquito breeding combined with the failure of the local authorities to respond on time.

### Late Start of the Rainy Season

**Fig. 1.**
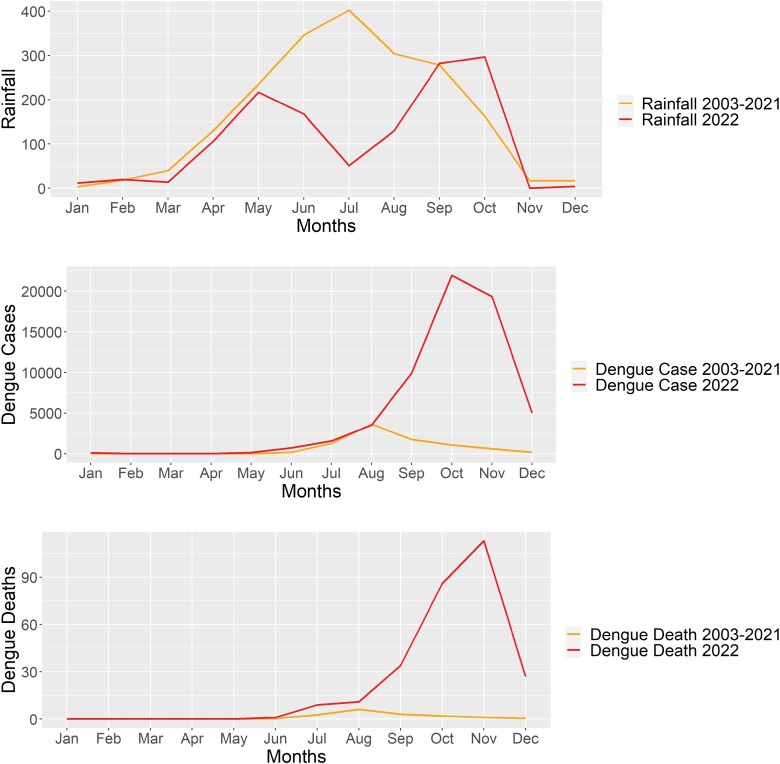
Top: The monthly average rainfall (millimeter) of the year 2022 in compared to the period of 2003–2021 recorded by the Bangladesh Meteorological Department in Mirpur, Dhaka, Bangladesh. Middle: The monthly average number of dengue cases reported for the period 2003–2021 and monthly total cases reported in 2022. Bottom: The monthly average recorded deaths for the period 2003–2021 is compared to the monthly total recorded death in 2022.

Bangladesh is in general a wet country receiving an annual 2,200 ml of rainfall (World Bank 2021). Rainfall is driven by the southwest monsoon which originates from the Indian Ocean and carries moisture and heat (World Bank 2021). Usually, rain starts in May and reaches its peak in July in Bangladesh (Fig. 1). The summer also coincides with the rainy season (April–September). The colder months (winter) including November to February are usually very dry with limited rainfall. In 2022, 297 mm of rainfall was recorded in the month of October, which is 133 mm higher than the monthly average rainfall for October for the period 2003–2021 (Table 1 and Fig. 1). This unusual rainfall facilitates the late start of the outbreak and intensifies the cases late in the season. Earlier in 2019, the unusually higher number of cases was related to an earlier start of the rainy season which allowed additional 2–3 months of mosquito breeding season (Ahsan et al. 2021). Furthermore, the year 2022 was relatively warmer, that is, compared to the monthly mean temperature of the period 2003–2021, in each month the temperature of 2022 was higher except in February, resulting in an additional 0.71 °C warmer year than the mean annual temperature of past 20 yr (Table 1). Temperature plays a vital role in the biological parameters of insects including biting rate, extrinsic incubation period, and vector survival rate. While higher temperatures could shorten the life span of the insects, it shortens the biting rate and extrinsic incubation period with a cumulative effect of faster transmission (Haider et al. 2017, Haider 2018). Thus, Bangladesh’s recent outbreak is associated with unusual weather patterns which are likely a consequence of global climate change.

**Table 1.** The mean monthly temperatures (°C), rainfall (millimeter), dengue cases, and recorded deaths caused by dengue virus infection for the period 2003–2021 and the year 2022

Months	Mean temp 2022 (°C)	Mean temp 2003–2021 (°C)	Monthly total rainfall 2022 (mm)	Monthly total rainfall 2003–2021 (mm)	Monthly total dengue cases 2022	Monthly mean dengue cases 2003–2021	Monthly total dengue deaths 2022	Monthly average dengue deaths 2003–2021
Jan	20.20	19.14	12.0	3.35	126	21.89	0	0.00
Feb	21.60	22.85	20.0	18.73	20	7.74	0	0.00
Mar	28.45	27.02	14.0	40.24	20	7.05	0	0.00
Apr	30.05	29.23	106.0	129.93	23	12.32	0	0.11
May	29.45	29.67	217.0	234.88	163	27.74	0	0.00
June	30.20	29.71	168.0	345.55	737	187.11	1	0.37
Jul	31.00	29.44	51.0	402.48	1571	1281.21	9	2.74
Aug	30.80	29.64	130.0	304.19	3521	3629.68	11	6.26
Sep	29.95	29.52	282.0	278.35	9911	1766.21	34	3.11
Oct	28.80	28.29	297.0	163.21	21932	1068.68	86	2.00
Nov	25.90	24.81	0.0	17.02	19334	626.11	113	1.11
Dec	22.30	20.96	4.0	17.88	5024	188.00	27	0.47
Average (total for “rainfall”)	27.39	26.69	1301.0	1955.81	5198.5	735.3	23.41	1.34

### Introduction of New Serotype DEN-4

Between 2013 and 2018, DENV-2 has been the dominant serotype with a moderate number of DENV-1 cases detected in Bangladesh. DENV-3 was introduced in late 2018 and was the dominant serotype (91.9%) of 2019 outbreaks (Rahim et al. 2021, Shirin et al. 2022) (Table 2). In 2021, only DENV-3 was detected in the country (Rahim et al. 2021). DENV-4 has not been recorded in Bangladesh since 2013. A recent report from the WHO Bangladesh office showed that the DENV-4 has reappeared in the country in 2022 with the absence of the serotype in the past few years (WHO-Bangladesh 2022) (Table 2). Bangladesh Ministry of Health and Family Welfare’s Planning, Monitoring and Research Operational Plan published a summary of ongoing research projects including circulating serotypes of DENV in Bangladesh. The research shows that DENV-4 re-emerged in the capital city Dhaka after the last reported case of the serotype in 2002 (Shirin Tahmina 2022). Institute of Epidemiology, Disease Control, and Prevention (IEDCR) detected DENV-4 in 11% (*n* = 13) samples, and DENV-3 in 89% (*n* = 110) samples and with no other serotype being detected in 2022 (Personal communication: Dr Manjur Hossain Khan, Lead, virology lab, IEDCR and co-author of this article) (Shirin Tahmina 2022). Although the sample size was not enough it will be interesting to see whether the DENV-4 replaces DENV-3 and other serotypes. The serotype DENV-4 might cause another large outbreak in 2023 and onwards if enough control measures are not taken. The source of DENV-4 from where the virus is introduced is not well known however, it may have been introduced from neighboring countries where that serotype is circulating for some time (Gowri Sankar et al. 2021, Nonyong et al. 2021, Ur Rehman et al. 2022). As the DENV-4 serotype was not detected in the country for more than 10 yr, the exposure increased infection susceptibility to a large naïve population. Many people were already infected with other serotypes (80% seroprevalence in Dhaka city [Salje et al. 2016]) and thus infection with DENV-4 increased the risk of developing secondary dengue infection. Many deaths recorded in 2022 might be an indication of frequent secondary infection with the DENV-4 serotype in the country.

**Table 2.** The serotypes of dengue virus circulating in Bangladesh since 2013 (adapted from Bangladesh’s Institute of Epidemiology Disease Control and Research [IEDCR] and World Health Organization–Bangladesh Office) and several peer-reviewed articles (Muraduzzaman et al. 2018, Institute of Epidemiology Disease Control and Research 2019, Ahsan et al. 2021, Rahim et al. 2021, Shirin et al. 2022, WHO-Bangladesh 2022)

Years	Serotypes of DENV
2013–2016	DENV2 (predominant) followed by DENV1
2017	DENV2 (predominant) followed by DENV1
2018	DENV2 (predominant) followed by DENV2 and DENV1 and co-detection DENV2 & DENV3 and DENV1 & DENV3(few cases)
2019	DENV3 (predominant) followed by co-detection of DENV2 & DENV3 and DENV1 & DENV3 (few cases)(Shirin et al. 2019, 2022, Rahim et al. 2021)
2020	DENV3 (predominant)
2021	DENV-3 prominent (100%) (Rahim et al. 2021)
2022	DENV-3 and DENV 4 (WHO-Bangladesh 2022). DENV 3 predominant (89%) with re-emergence of DENV4 (11%). (Shirin Tahmina 2022) and Personal Communication: Dr Manjur Hossain Khan, IEDCR, Dhaka, Bangladesh

### Post-pandemic Returning to Normalcy

The COVID-19 pandemic has disrupted the health system of many low- and middle-income countries and the health system in Bangladesh has been under significant stress (al Sattar et al. 2022). Public health and local government authorities have invested their best efforts to limit COVID-19 transmission and to distribute vaccines. The control of Aedes mosquitoes during the post-pandemic returning to normalcy was therefore not a top priority. Although Bangladesh was successful in distributing COVID-19 vaccines to its large population, limited resources in the local government meant other health issues were not adequately addressed, that is, the control of mosquito populations. Stagnant and clear water around the construction sites was identified as the most suitable place for Aedes mosquito breeding in Bangladesh by a survey by the Directorate General of Health Services (Helemul Alam 20221). According to a survey, flooded basements, plastic barrels, and water tanks at construction sites were the most prolific breeding sites (Helemul Alam 20221). Due to the COVID-19 pandemic, construction and house-building activities had been interrupted or slowed from 2020 to 2021. As the lockdown and other non-pharmaceutical restriction measures were removed and life returned to normalcy, construction works started in full swing to make up for the lost work in the previous 2 yr. Construction sites likely provided extra opportunities for mosquito breeding in the country and increased human exposure to mosquitoes facilitates the spreading of the virus in the community.

### Bangladesh’s Public Health Response

Bangladesh’s MOHFW has taken several initiatives (WHO-Bangladesh 2022). The authorities repurposed 6 COVID-19 dedicated hospitals in Dhaka city for treating dengue patients (WHO-Bangladesh 2022). In most tertiary care hospitals (medical colleges/university hospitals), the authorities established a dedicated dengue ward. The WHO and MOHFW distributed more than 250,000 rapid diagnostic kits to subdistrict hospitals (WHO-Bangladesh 2022). The city corporation arranged awareness programs and informed the building owners and construction sites to prevent water collection and stagnation. The local government engineering department led mosquito control activities including the destruction of mosquito breeding sites and the spraying of adulticides insecticides (WHO-Bangladesh 2022).

### Public Health Challenges

Controlling Aedes mosquitoes and dengue virus, especially in tropical countries is undeniably difficult. Understanding the mosquito life cycle and the disease ecology, is a vital part of the mosquito control program. Besides dengue, Aedes mosquitoes are the vector of a wide range of human and animal pathogens including flaviviruses (Japanese Encephalitis, West Nile, and yellow fever), alphaviruses (chikungunya, Eastern Equine Encephalitis, Ross River virus), and bunya viruses (Rift valley fever, LaCrosse virus) (Benedict et al. 2007) and all these viruses pose a public health threat to Bangladesh.

Insecticide resistance has become more prevalent in recent years with at least one of the 4 major classes of insecticides reported ineffective in 80% of malaria-endemic countries (Di Rocco et al. 1997). Scientists identified resistance to permethrin for *Aedes albopictus* in Bangladesh (Al-Amin et al. 2022). Permethrin is currently being used as an adulticide by the local authorities in Bangladesh (Ahsan et al. 2021, Al-Amin et al. 2022). No promising new insecticides are in the pipeline which is likely to lead to the widespread use of indiscriminate pesticides in the future, leading to a rapid decline of non-target insect populations, and inducing growing resistance to insecticides. Although there is a vaccine being licensed, the efficacy and effectiveness of the vaccines are still controversial. Another successful vaccine is yet to be discovered, but the antibody-dependent enhancement (ADE) related to different serotypes of dengue virus is a real obstacle to using such vaccines in endemic countries (Wang et al. 2017). The endemic countries need to engage multiple sectors and departments and keep working all year round to control the mosquito population and breeding sites. Engaging community volunteers and training them to destroy mosquito habitats effectively and regularly should be prioritized (Ahsan et al. 2021).

## Conclusions

Bangladesh recorded the highest-ever reported annual deaths from dengue virus infection in 2022. The 2022 outbreak is characterized by late onset and late resurgence of dengue cases. Late rainfall in September and October facilitates mosquito breeding and intensification of the outbreaks. Being one of the warmest years in the past 20 yr, the increased temperature of 2022 facilitated the faster transmission of the dengue virus in the community. The new serotype DENV-4 for which most people are immunologically naïve likely resulted in the rapid spread of the virus. Post-COVID-19 pandemic return to normalcy, especially in the construction industry, facilitates mosquito breeding. The local public health agencies and health systems which were already under significant pressure because of the COVID-19 pandemic were unable to respond in time to control the mosquito population. The recent 2 large outbreaks in 2019 and 2022 were associated with unusual weather patterns (early rainfall in 2019 and late rainfall in 2022) which were likely an effect of global climate change. Community engagement and year-round destruction of Aedes mosquito habitats should be prioritized to control the dengue epidemic in Bangladesh.

## Data Availability

All the dengue data presented in this manuscript are publicly available on Bangladesh’s Ministry of Health and Family Welfare’s Directorate General of Health Services website (https://dghs.gov.bd/). The meteorological data were purchased from Bangladesh Meteorological Department and are restricted to use for research purpose only and anyone interested in these data can request Bangladesh Meteorological Department (https://live3.bmd.gov.bd/).
